# A deleterious role for Th9/IL-9 in hepatic fibrogenesis

**DOI:** 10.1038/srep18694

**Published:** 2016-01-05

**Authors:** Shan-yu Qin, Dong-hong Lu, Xiao-yun Guo, Wei Luo, Bang-li Hu, Xiao-li Huang, Mei Chen, Jia-xu Wang, Shi-Jia Ma, Xian-wen Yang, Hai-xing Jiang, You Zhou

**Affiliations:** 1 Department of Gastroenterology, the First Affiliated Hospital of Guangxi Medical University, Nanning 530021, China; 2 Systems Immunity University Research Institute, Cardiff University School of Medicine, Heath Park, Cardiff, UK; 3 Minerva Foundation Institute for Medical Research, Helsinki, Finland

## Abstract

T helper 9 (Th9) cells, a recently recognized Th cell subset, are involved in autoimmune diseases. We aimed to investigate the role of Th9/interleukin-9 (IL-9) in the pathogenesis of hepatic fibrosis. Th9 and Th17 cells were quantified in chronic hepatitis B (CHB) patients with hepatic fibrosis, HBV-associated liver cirrhosis (LC) patients and healthy controls (HC). The percentages of Th9 and Th17 cells, concentrations of IL-9 and IL-17, as well as expression of IL-17, TNF-α, IL-6, IL-4, IL-21, TGF-β1 and IFN-γ were significantly increased in plasma of CHB and LC patients compared with those in HC. Splenic Th9 and Th17 cells, plasma concentrations and liver expression of IL-9 and IL-17A were significantly elevated in mice with hepatic fibrosis compared with controls. Neutralization of IL-9 in mice ameliorated hepatic fibrosis, attenuated the activation of hepatic stellate cells, reduced frequencies of Th9, Th17 and Th1 cells in spleen, and suppressed expression of IL-9, IL-17A, IFN-γ, TGF-β1, IL-6, IL-4 and TNF-α in plasma and liver respectively. Our data suggest a deleterious role of Th9/IL-9 in increasing hepatic fibrosis and exacerbating disease endpoints, indicating that Th9/IL9 based immunotherapy may be a promising approach for treating hepatic fibrosis.

Hepatic fibrosis, a major consequence of chronic liver injury, has a wide range of causes including viral infection, excess intake of alcohol, fat deposition and autoimmune reactions. The status of hepatic fibrosis is characterized by abnormal accumulation of extracellular matrix components and activation of hepatic stellate cells (HSCs)^1^. Accumulating data suggest that infiltrating CD4^+^ T lymphocytes including T helper (Th) cells and regulatory (Tregs) cells play important roles in mediating liver inflammation and fibrosis progression^2^,^3^,^4^,^5^,^6^,^7^. It has become evident that several major subsets of CD4^+^ Th cells such as Th1, Th2 and Th17 regulate the pathogenesis of hepatic fibrosis^6^,^7^,^8^. However, the precise role of different Th cell subsets and related fundamental mechanisms in the development of hepatic fibrosis remain unclear.

As a recently recognized Th cell subset characterized by secreting large quantities of interleukin-9 (IL-9), Th9 cells are involved in a broad range of autoimmune disorders and allergic inflammation^9^,^10^,^11^. Recently, there has been a rapidly growing interest in the role of Th9 cells since they prominently modulate host responses via interacting with different T cell populations^12^,^13^,^14^. The cells exert either pro- or anti-inflammatory activities by regulating the development of Treg and/or Th17 cells^15^,^16^. Furthermore, IL-9 induces immunosuppression controlled by Tregs and mast cells, resulting in the tolerance to environmental stresses^10^,^11^,^17^. As a pleiotropic cytokine, IL-9 both positively and negatively regulates immune responses.

Th17 cells, defined by their secretion of interleukin-17 (IL-17), play a profound role in the development of hepatic fibrosis. An imbalance between Th17 and Treg cells promotes liver fibrosis via HSC activation^18^,^19^. In contrast to Th17 cells, little is known about the role of Th9 cells in the pathogenesis of hepatic fibrosis. In the present study, we analyzed the association between Th9/IL-9 and liver fibrosis in patients with either LC (liver cirrhosis) or CHB (chronic hepatitis B). We further examined the role of the endogenous IL-9 in hepatic fibrosis and its relationship with other relevant cytokines, including IL-17A, IFN-γ, TGF-β1, IL-6, IL-4, IL-21 and TNF-α in response to hepatic fibrosis, by neutralizing IL-9 in a mouse model. We describe for the first time that Th9/IL9 has a deleterious role that leads to increased hepatic fibrosis and an exacerbated disease endpoints.

## Results

### Alterations of plasma Th9/IL-9 and Th17 in chronic hepatitis B (CHB) and HBV-associated liver cirrhosis (LC) patients

**Figure 1:**
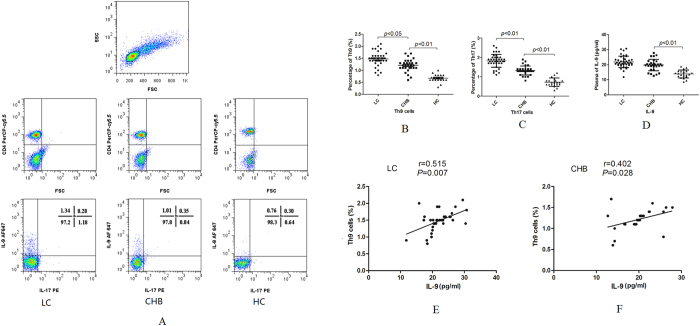
Plasma Th9, Th17 cells and IL-9 in patients with liver cirrhosis (LC), chronic hepatitis B (CHB) associated liver fibrosis and healthy controls (HC). (**A**) Characterization of plasma CD4^+^ Th-cell populations by flow cytometry in LC, CHB and HC subjects. Total lymphocytes were measured (panels in the first row) and CD4^+^ T cells were analyzed (panels in the second row). The percentages of Th9 cells (IL-9^+^ CD4^+^ cells) and Th17 (IL-17^+^CD4^+^ cells) in LC, CHB patients and HC are shown in the third row. (**B**–**C**) Comparison of percentages of Th9 and Th17 cells between LC, CHB patients and HC. (**D**) Comparison of plasma levels of IL-9 between LC, CHB patients and HC. (**E**) Correlation between levels of IL-9 and percentages of Th9 cells in plasma of LC patients. (**F**) Correlation between levels of IL-9 and percentages of Th9 cells in plasma of CHB patients.

Plasma Th9 and Th17 cells were determined by flow cytometry based on cytokine patterns (Fig. 1A). The percentages of plasma Th9 cells were significantly elevated in patients with LC or CHB compared with healthy controls (HC) (*p* < 0.01) (Fig. 1B). Consistently, the percentages of Th17 cells were significantly increased in both patient groups compared with HC (*p* < 0.01, Fig. 1C). Plasma levels of IL-9 were remarkably higher in patients with CHB or LC than in HC (*p* < 0.01, Fig. 1D). Furthermore, IL-9 levels were positively correlated with Th9 percentages in both LC (r = 0.515, *p* = 0.007, Fig. 1E) and CHB patients (r = 0.402, *p* = 0.028, Fig. 1F).

### Correlations of Th9 cells with laboratory parameters

We did not find significant associations between the percentages of Th9 cells and levels of ALT (r = −0.181, *p* = 0.433), total bililubin (r = 0.008, *p* = 0.973) and copy numbers of HBV DNA (r = 0.237, *p* = 0.302) in plasma of patients with CHB. Similarly, no significant correlations were identified between the percentages of Th9 cells and those parameters in plasma of patients with LC (ALT: r = 0.096, *p* = 0.578; total bililubin: r = −0.185, *p* = 0.279; copy numbers of HBV DNA: r = −0.062, *p* = 0.718).

### Plasma levels of cytokines in patients and controls

**Figure 2:**
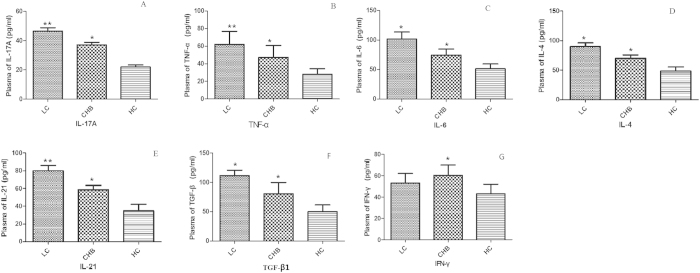
Comparisons of plasma levels of (**A**) IL-17A, (**B**) TNF-α, (**C**) IL-6, (**D**) IL-4, (**E**) IL-21, (**F**) TGF-β1 and (**G**) IFN-γ between LC, HB and HC groups. *p-values < 0.05, **p < 0.01.

ELISA showed that plasma levels of IL-17A, TNF-α, IL-6, IL-4, IL-21 and TGF-β1 were significantly increased in LC patients compared with CHB patients and HC (Fig. 2A–F). In addition, plasma levels of IL-17A, TNF-α, IL-6, IL-4, IL-21 and TGF-β1 were significantly higher in CHB patients than in HC (Fig. 2A–F). Plasma levels of IFN-γ were also higher in CHB group than in LC group and HC (Fig. 2G). In contrast, no significant difference of IFN-γ levels was observed between LC patients and HC (Fig. 2G).

### The development of hepatic fibrosis in CCL4 treated mice

**Figure 3:**
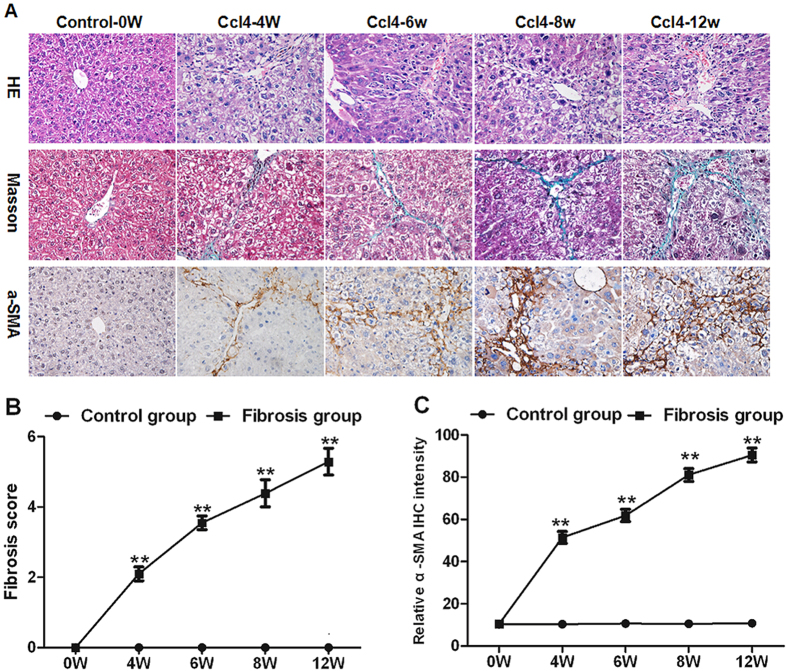
The severity of liver fibrosis in CCl4 administrated mice from 0 to 12 weeks. (**A**) Histology was evaluated by H&E staining. Fibrillar collagen deposition was assessed by Masson staining (original magnification, ×400). Activated HSCs in liver sections were quantified by immunohistochemical staining of alpha-smooth muscle actin (α-SMA) (original magnification, ×400). (**B**) Change of liver fibrosis score from 0 to 12 weeks after CCl4 administration. (**C**) Change of relative intensity of α-SMA from 0 to 12 weeks. The mean number of α-SMA-positive cells in five ocular fields per specimen was assessed as a percentage area at 400× magnification. **p <  0.01 vs. control subgroups sacrificed at the same time point. Data are shown as mean ± SD.

The administration of CCl4 to mice caused significant hepatic inflammation, including hepatocyte ballooning and necrosis. Necroinflammation, perisinusoidal and portal fibrosis were formed at 8^th^ week. In addition, irregular necrosis and bridging fibrosis emerged at this time point (Fig. 3A). The liver fibrosis, as shown by Masson staining, grew more severe from 0 to 12 weeks (panels in the second row, Fig. 3A). As an indicator of the severity of hepatic fibrosis, the Ishak fibrosis score increased from 0^th^ to 12^th^ week, and peaked at 12^th^ week (Fig. 3B).

The activation of HSCs, a major event during the development of liver fibrosis, could be monitored by expression of α-smooth muscle actin (α-SMA). The intensity of α-SMA staining increased from 0 to 12 weeks (panels in the third row, Fig. 3A,C). α-SMA spread to the portal area of liver tissue at the 12^th^ week, indicating the activation of HSCs was enhanced (panels in the third row, Fig. 3A).

### Splenic Th9 and Th17 cells in mice with hepatic fibrosis

**Figure 4:**
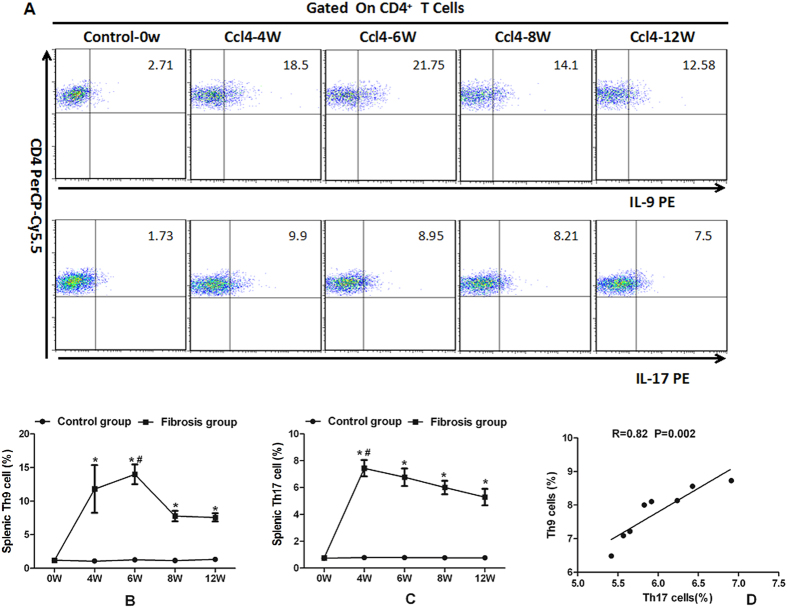
Percentages of splenic Th9 and Th17 cells significantly increased in mice with hepatic fibrosis. (**A**) Characterization of splenic Th9 and Th17 cells by flow cytometry in CCl4 administrated mice at the 0^th^, 4^th^, 6^th^, 8^th^ and 12^th^ week. (**B**) Change of percentages of splenic Th9 cells in mice at different time points. (**C**) Change of percentages of splenic Th17 cells in mice at different time points. *p < 0.05 *vs.* controls sacrificed at the same time point; ^#^*p* < 0.05 *vs.* other subgroups with fibrosis. Data are presented as mean ± SD. (**D**) Correlation between splenic Th9 and Th17 cells in mice with liver fibrosis at the 8^th^ week.

There were few Th9 and Th17 cells in murine liver tissue, which were difficult to be detected by flow cytometry^20^. Thus we quantified those cells in splenic tissue. After CCl4 treatment, splenic Th9 and Th17 cells at 0, 4, 6, 8 and 12 weeks were measured by flow cytometry (Fig. 4A). The percentages of splenic Th9 (IL-9^+^CD4^+^) cells increased during the first 6 weeks and peaked at the 6^th^ week (*p* < 0.05, Fig. 4B) while the Th17 (IL-17^+^CD4^+^) percentages increased during the first 4 weeks and peaked at the 4^th^ week (*p* < 0.05, Fig. 4C). Neither Th9 nor Th17 percentages were altered in the controls from 0 to 12 weeks. Splenic Th9 and Th17 percentages were both higher in mice with liver fibrosis than in controls (*p* < 0.05, Fig. 4B,C). Notably, there was a strong correlation between splenic Th9 and Th17 cells in mice with hepatic fibrosis (r = 0.82, *p* = 0.002, Fig. 4D).

### Plasma concentrations and liver mRNA of IL-9 and IL-17A in mice with hepatic fibrosis

**Figure 5:**
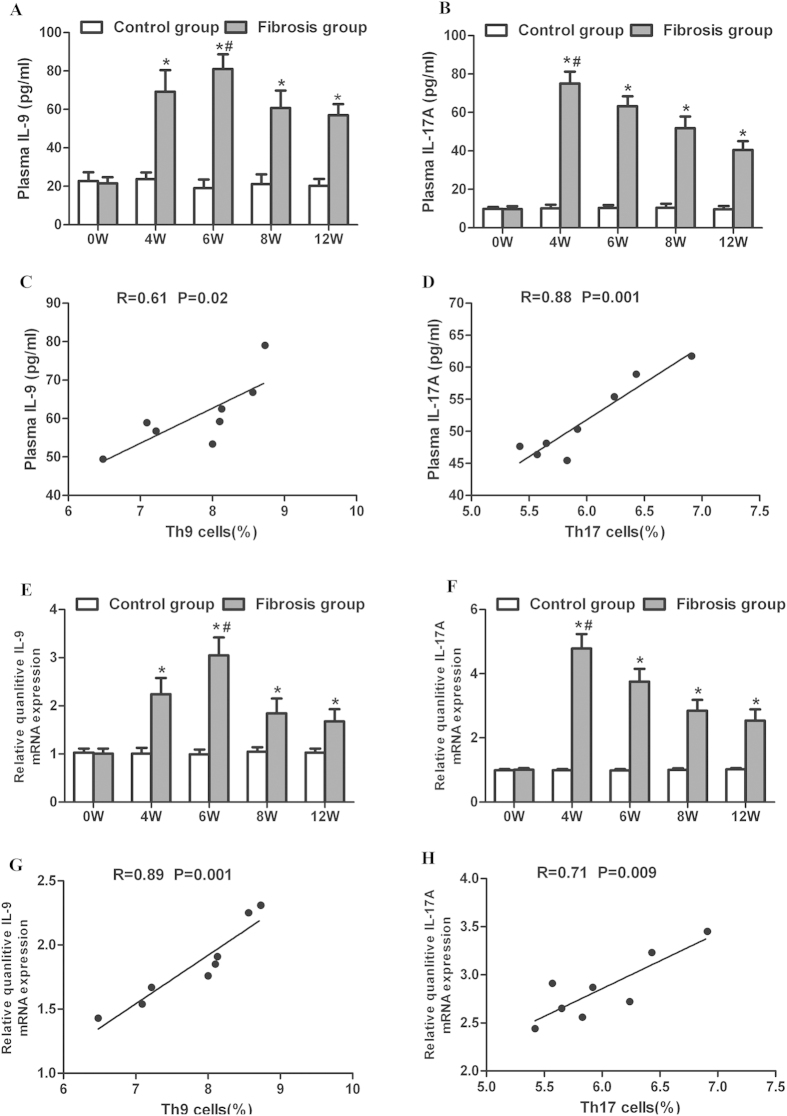
plasma levels and liver expression of IL-9 and IL-17A in mice with hepatic fibrosis. (A–B) Plasma levels of IL-9 and IL-17A in CCl4 administrated mice at the 0^th^, 4^th^, 6^th^, 8^th^ and 12^th^ week. (**C**) Correlation between plasma IL-9 levels and splenic Th9 percentages in the mouse model of hepatic fibrosis at the 8^th^ week. (**D**) Correlation between plasma IL-17 and splenic Th17 cells in the mouse model of hepatic fibrosis at the 8^th^ week. (**E–F**) Liver expression of IL-9 and IL-17A in CCl4 administrated mice at the 0^th^, 4^th^, 6^th^, 8^th^ and 12^th^ week. (**G**) Correlation between liver expression of IL-9 and percentages of splenic Th9 cells in mice at the 8^th^ week. (**H**) Correlation between liver expression of IL-17 and percentages of splenic Th17 cells in mice at the 8^th^ week. *p < 0.01 *vs.* controls sacrificed at the same time point. ^#^*p* < 0.05 *vs.* other subgroups with fibrosis. Data are presented as mean ± SD.

After CCl4 administration, plasma concentrations of IL-9 were elevated, peaking at the 6^th^ week (*p* < 0.05, Fig. 5A). Similarly, plasma concentrations of IL-17A increased, peaking at the 4^th^ week (*p* < 0.05, Fig. 5B). Plasma concentrations of IL-9 and IL-17A were both remarkably higher in mice with liver fibrosis than in controls (Fig. 5A,B). Furthermore, plasma levels of IL-9 and IL-17A were significantly correlated with the percentages of splenic Th9 (Fig. 5C) and Th17 cells (Fig. 5D) in mice with hepatic fibrosis, respectively.

Liver mRNA levels of IL-9 were significantly raised and peaked at the 6^th^ week after treatment with CCl4 (*p* < 0.05, Fig. 5E). Liver mRNA levels of IL-17A increased and peaked at the 4^th^ week (*p* < 0.05, Fig. 5F). The mRNA levels of IL-9 and IL-17A were higher in mice with liver fibrosis than in controls (Fig. 5E,F). In addition, there were strong associations between liver IL-9 and IL-17 mRNA levels and percentages of splenic Th9 (r =  0.89, *p* = 0.001, Fig. 5G) and Th17 cells (r = 0.71, *p* = 0.009, Fig. 5G), respectively.

### Neutralization of IL-9 attenuates the severity of hepatic fibrosis

**Figure 6:**
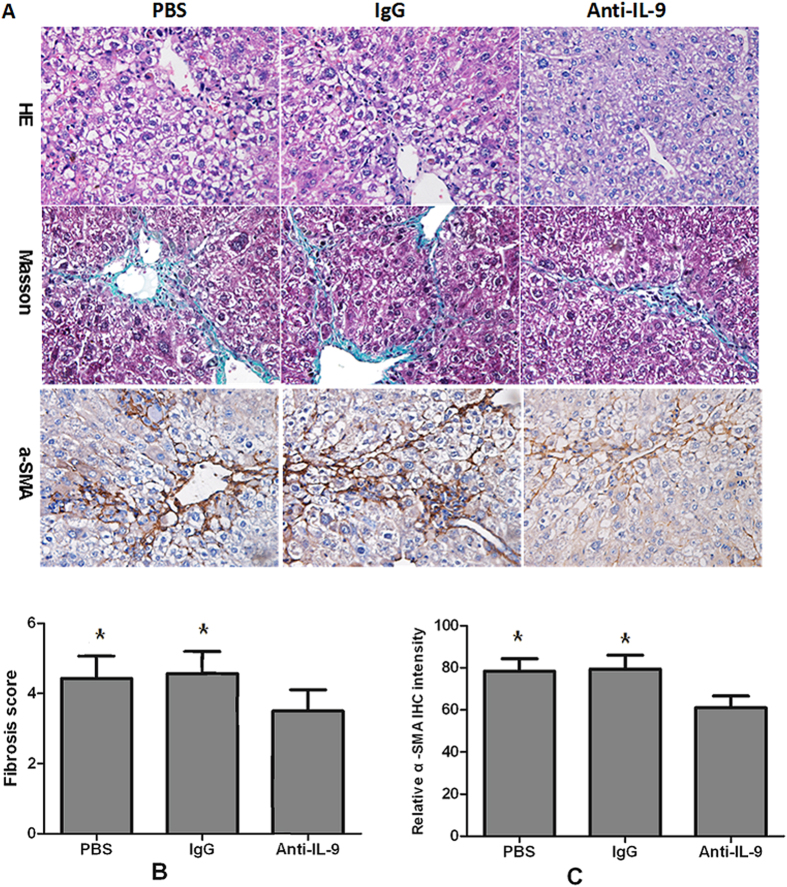
anti-IL-9Ab attenuated the severity of hepatic fibrosis. (**A**) Histology was assessed by H&E staining. Fibrillar collagen deposition was evaluated by Masson staining (original magnification, ×400). Activated HSCs in liver sections were quantified by immunohistochemical staining of alpha-smooth muscle actin (α-SMA) (original magnification: ×400). (**B**) Comparison of Ishak fibrosis score between anti-IL-9Ab, PBS and IgG treated groups. (**C**) Comparison of relative intensity of α-SMA in anti-IL-9Ab, PBS and IgG treated groups. **p < 0.01 compared with control PBS or IgG treated mice. Data are shown as mean ± SD.

To investigate the role of IL-9 in modulating hepatic fibrosis *in vivo*, we treated the mouse model of hepatic fibrosis with IL-9-neutralizing antibodies (anti-IL-9Ab) by intraperitoneal (IP) injection. 8 weeks treatment with anti-IL-9Ab remarkably attenuated hepatic inflammation, necrosis and fibrosis in mice with hepatic fibrosis compared with control IgG-treated and PBS-treated mice (Fig. 6A). Mice treated with anti-IL-9Ab had a significant lower liver fibrosis score than IgG-treated and PBS-treated controls (*p* < 0.05, Fig. 6B). Consistently, the number of α-SMA positive hepatic cells was significantly reduced in anti-IL-9Ab treated mice (*p* < 0.05, Fig. 6C). These data suggested that the development of hepatic fibrosis was suppressed in the absence of IL-9, which was associated with a decreased number of α-SMA positive cells.

### Neutralization of IL-17 attenuates the severity of hepatic fibrosis

Similar to the impact of anti-IL-9Ab treatment on hepatic fibrogensis, IP injection of IL-17-neutralizing antibodies (anti-IL-17Ab) for 8 weeks significantly attenuated hepatic inflammation, necrosis and fibrosis in mice with hepatic fibrosis compared with controls (Fig. S1A). Anti-IL-17Ab-treated mice had a lower liver fibrosis score and a smaller number of α-SMA positive hepatic cells as compared with controls (Fig. S1B-C). Neither the liver fibrosis score nor the number of α-SMA positive hepatic cells differed significantly between anti-IL-9Ab and anti-IL-17Ab treated mice (data not shown).

### Treatment with anti-IL-9Ab reduces percentages of splenic Th9, Th17 and Th1 cells in mice with hepatic fibrosis

**Figure 7:**
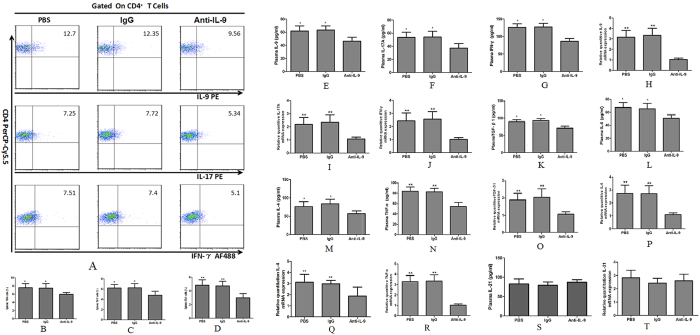
The percentages of splenic Th9, Th17 and Th1 cells, plasma secretion and liver expression of the relevant cytokines in IL-9 neutralized mice with liver fibrosis. (**A**) Characterization of splenic Th9 and Th17 cells in fibrosis mice treated with anti-IL-9Ab, PBS and IgG. (**B**–**D**) The percentages of splenic Th9, Th17 and Th1 cells in anti-IL-9Ab, IgG and PBS treated groups. (**E**–**G**) Plasma levels of IL-9, IL-17A and IFN-γ in anti-IL-9Ab, IgG and PBS treated groups. (**H**–**J**) The liver mRNA levels of IL-9, IL-17A and IFN-γ in anti-IL-9Ab, PBS and IgG treated groups. (**K**–**N**) The plasma levels of TGF-β1, IL-6, IL-4 and TNF-α in anti-IL-9Ab, IgG and PBS treated groups. (**O**–**R**) The liver mRNA levels of TGF-β1, IL-6 IL-4 and TNF-α in anti-IL-9Ab, IgG and PBS treated groups. (**S**) The plasma levels of IL-21 in anti-IL-9Ab, IgG and PBS treated groups. (**T**) The liver mRNA levels of IL-21 in anti-IL-9Ab, IgG and PBS treated groups. **p <  0.01 compared with control PBS or IgG treated mice. Data are presented as mean ± SD.

After treating fibrosis mice with anti-IL-9Ab, control PBS or IgG for 8 weeks, we gated splenic Th9, Th17 and Th1 subsets by flow cytometry (Fig. 7A). We observed significantly decreased percentages of Th9, Th17 and Th1 cells in anti-IL-9Ab treated mice than in controls (Fig. 7B–D). Plasma levels of IL-9, IL-17A and IFN-γ were significantly lower in anti-IL-9Ab treated as compared with PBS or IgG treated mice (Fig. 7E–G). Consistently, we found liver mRNA expression of IL-9, IL-17A and IFN-γ significantly decreased in the anti-IL-9Ab treated mice (Fig. 7H–J).

### Treatment with anti-IL-9Ab decreases plasma and liver mRNA levels of specific cytokines

To better understand the role of IL-9 in regulating inflammatory response, we measured levels of inflammatory cytokines in plasma and liver tissues of mice with liver fibrosis. Anti-IL-9Ab treatment sharply decreased plasma concentrations of TGF-β1, IL-6, IL-4, and TNF-α in a mouse model of liver fibrosis (Fig. 7K–N). Consistently, liver mRNA expression of TGF- β1, IL-6, IL-4, and TNF-α were significantly reduced in the anti-IL-9Ab treated mice compared with PBS or IgG treated controls (Fig. 7O–R). In contrast, neither plasma concentrations nor liver mRNA expression levels of IL-21 showed any difference between anti-IL-9Ab, PBS and IgG treated mice (Fig. 7S–T).

### Treatment with anti-IL-17Ab decrease plasma and liver mRNA levels of specific cytokines

After treatment with anti-IL-17Ab for 8 weeks, the percentages of splenic Th9, Th17 and Th1 cells were reduced in mice with liver fibrosis (Fig. S2A-C). Consistently, plasma concentrations and liver mRNA levels of IL-9, IL-17A and IFN-γ significantly decreased in the anti-IL-17Ab treated mice as compared with PBS or IgG treated controls (Fig. S2D-I). Additionally, specific cytokines including TGF-β1, IL-6, IL-4, IL-21 and TNF-α showed significantly reduced levels in both plasma (Fig. S2J-N) and liver tissues of the anti-IL-17Ab treated mice as compared with controls (Fig. S2O-S). To be noted, plasma and liver mRNA levels of IL-21 significantly decreased in the IL-17-neutralized (Fig. S2M and S2S) but not in the IL-9-neutralized mice (Fig. 7S,T), indicating a regulatory difference between IL-9 and IL-17 in the pathogenesis of liver fibrosis.

## Discussion

Increasing evidence suggests that several CD4^+^ Th subsets and their specific cytokines such as Th1/IFN-γ and Th17/IL-17 play crucial roles in the pathogenesis of hepatic fibrogenesis^21^. However, the role of Th9/IL-9 cells in hepatic fibrosis is unknown. In this study, we observed significantly increased percentages of Th9 and levels of IL-9 in plasma of CHB and LC patients with liver fibrosis compared with HC. Subsequently, we identified a positive correlation between plasma Th9 and Th17 frequencies in CHB and LC patients with liver fibrosis. Furthermore, we found that percentages of splenic Th9 and Th17 cells, plasma concentrations and liver expression of both IL-9 and IL-17A were significantly higher in mice with hepatic fibrosis than in controls. The percentages of Th9/IL-9 cells significantly elevated in both patients and mice with liver fibrosis, suggesting that the microenvironment induced by liver fibrosis favors Th9 proliferation and IL-9 secretion. Importantly, we showed decreased percentages of Th9, Th17 and Th1 cells in spleen along with an attenuation of hepatic fibrosis in IL-9-neutralized mice, indicating endogenous IL-9 plays a deleterious role in hepatic fibrogenesis. Neutralization of IL-9 further suppressed expression of inflammatory cytokines including IL-9, IL-17A, IFN-γ, TGF-β1, IL-6, IL-4 and TNF-α in the mouse model of hepatic fibrosis, suggesting IL-9 may regulate the inflammatory responses to liver fibrosis.

Out study showed that treatment with anti-IL-9Ab and anti-IL-17Ab exerted similar effects on suppressing plasma secretion and liver expression of TGF-β1, IL-6, TNF-α, IL-4 and IL-9 in mice with liver fibrosis. We further delineated differences between IL-9 and IL-17 and their roles that played in liver fibrosis by using levels of IL-21 as an indicator. Neutralization of IL-17 reduced plasma concentration and liver expression of IL-21 but neutralization of IL-9 did not. These findings suggested that the endogenous IL-17 leads to increased hepatic fibrosis through regulating IL-21 while IL-9 may not. IL-21, an IL-2 family cytokine, is primarily produced by Th-17 cells. The role of IL-21 in Th17 differentiation is still under debating^22^,^23^. It has been reported that IL-21 inversely correlates with Th9 differentiation and IL-9 production^24^.

Th9/IL-9 cells are involved in a broad range of immune-mediated inflammatory diseases. Several studies showed that Th9 cells mediate the responses occurred in peripheral neuritis^9^, allergic inflammation^11^ and colitis^25^. A very recent study observed an increasing trend of serum IL-9 levels in only twenty LC patients compared with normal controls and CHB patients^26^. We found that the IL-9 neutralization ameliorated the development of hepatic inflammation, necrosis and fibrosis. Moreover, anti-IL-9Ab treatment markedly decreased the number of activated HSCs. These findings suggested that anti-IL-9Ab treatment ameliorated liver fibrosis by inactivating HSCs. Furthermore, we showed that anti-IL-9Ab treatment decreased numbers of Th subsets including Th9, Th17 and Th1, indicating that IL-9 may down-regulate the proliferation of Th9, Th17 and Th1 cells in response to hepatic fibrosis. To be noted, we showed that the treatment with IL-9 significantly reduced plasma levels and liver expression of IL-9, IL-17A, IFN-γ, TGF-β1, IL-6 and TNF-α in a fibrotic mouse model, which suggested that anti-IL-9Ab inhibited the responses to liver fibrosis through modulating the release of the cytokines including TGF- β1, IL-6 and TNF-α. In line with our findings, a study reported that IL-9 neutralized mice had not only lower spinal cord mRNA levels of IL-17, IFN-γ, IL-6 and TNF-α, but also decreased Th17 and Th1 response against autoimmune encephalomyelitis^27^. Similarly, one study reported that IL-9 neutralization markedly reduced expression of TGF-β, vascular endothelial growth factor and fibroblast growth factor 2 in lung tissue after prolonged ovalbumin exposure^28^.

Th17 cells are involved in the pathogenesis of immune-mediated tissue injury, including viral hepatitis^29^,^30^, autoimmune hepatitis^31^ and hepatic fibrosis^18^,^31^. Several *in vitro* studies presumed that a complex regulatory network might exist between Th9 and Th17 cells^32^,^33^. IL-9 could function as an autocrine growth factor that facilitates the expansion of Th17 cells^32^,^33^,^34^. We showed *in vivo* a positive correlation between Th9 and Th17 cells in a fibrotic mouse model, indicating the connection between those two Th cell subsets play a synergistic role in the development of hepatic fibrosis.

IL-17, mainly produced by Th17 cells, is a proinflammatory and fibrogenic cytokine. IL-17 signaling enhances the production of IL-1, IL-6 and TNF-α in inflammatory cells and increases the expression of a fibrogenic cytokine, TGF-β1^35^. In addition, IL-17 induces the production of collagen type I in HSCs by activating the STAT3 pathway^36^. We found that anti-IL-17Ab treatment alleviated liver fibrosis in mice. In line with our observation, several studies recently reported that knockout or blockade of endogenous IL-17 attenuated the development of liver injury and fibrosis^37^,^38^. Thus, IL-17 plays a determinant role in the progression of liver fibrosis.

Unlike IL-17, little is known about the role of IL-9 in fibrosis. Van den Brule reported that overexpression of IL-9 exacerbated airway fibrosis that was induced by the chronic instillation of an allergenic mold^39^. However, Arras *et al.* found that IL-9 reduced the lung fibrotic process induced by instillation of crystalline silica particles^40^. Additionally, IL-9 exerts pluripotent function in some specific pathways. For example, IL-9 was reported to mediate CCL11 expression in airway smooth muscle cells through the STAT3 pathway^41^. In atopic dermatitis patients, IL-9 was shown to regulate the IL-9-STIM1-ERK-IL-8 axis in keratinocyte^42^.

The differentiation of T cells into Th subsets such as Th9 and Th17 are largely dependent on the microenvironment made up by secreted cytokines. TGF-β has been classified as a regulatory cytokine that inhibits the cell proliferation. It regulates the differentiation of T cells into the Th1 and Th17 phenotypes in both human and murine^43^. TNF-α, IL-6 together with TGF-β prompts the generation of Th17 cells^44^,^45^, whereas TGF-β plus IL-4 induces a Th9 response^13^. Several studies showed that TGF-β together with IL-6 activated the pathway of Th17 differentiation^44^,^45^ while TGF-β together with IL-4 induced the differentiation of CD4^+^ T cells into Th9 cells^46^,^47^,^48^. IL-4 inhibits TGF-β-induced Foxp3 expression, which leads the differentiation of CD4^+^ T cells towards Th9 cells^46^,^47^,^49^ rather than Treg phenotype. A study reported that IFN-γ or IL-27 inhibited IL-9 secretion in human CD4^+^ T cells cultured with TGF-β and IL-4^48^. Inflammatory cytokines such as IL-1b, IL-6, IL-10, IFN-α, IFN-β and IL-21 enhance IL-9 expression and Th9 cells differentiation. On the other hand, blockade of IL-21 impairs Th9 cells differentiation enhanced by cytokines^48^,^49^.

## Conclusion

Our results demonstrate that both Th9 and Th17 cells are involved in the development of hepatic fibrosis *in vivo*. Treatment with anti-IL-9Ab attenuates HSC activation, inhibits the proliferation of splenic Th9, Th17 and Th1 cells, and suppresses expression and secretion of IL-9, IL-17A, IFN-γ, TGF-β, IL-6, IL-4 and TNF-α rather than IL-21 thereby ameliorates hepatic fibrogenesis. Altogether, Th9/IL9 has a deleterious role that leads to increased hepatic fibrosis. Th9/IL-9 may serve as a potential target for therapeutic intervention of hepatic fibrosis.

## Materials and Methods

### Patients and controls

The subjects were recruited at the First Affiliated Hospital of Guangxi Medical University, China from December 2012 to February 2014. We excluded patients if they were <18 or >65 years old; or had severe complications of liver disease; or underwent systemic antibiotics treatment, or any immunomodulatory or immunosuppressive therapy during the 6 month prior to sampling. We also excluded patients with co-infections of hepatitis C, D virus, HIV infections or had a history of alcohol abuse or evidence of autoimmune liver disease. All subjects underwent clinical, radiological or histological diagnoses that were performed in line with international diagnostic criteria. The study protocol was approved by the ethics committee of the First Affiliated Hospital of Guangxi Medical University. The methods used in relation with humans were carried out in accordance with the approved guidelines. Each participant signed informed consent after being explained the nature and potential risks of the study. In the eligible subjects (n = 90), 30 patients had chronic hepatitis B (CHB) with hepatic fibrosis, 40 had HBV-associated cirrhosis and 20 were healthy individuals. Blood samples were taken from all the subjects. The clinical characteristics of eligible subjects are shown in Table 1.

### Animal

Specific pathogen free male BALB/c mice aged 6 week weighing 18–20 g were purchased from the Laboratory Animal Center (Guangxi Medical University, China, No. SCXKG 2010-0002). Animals were kept in the pathogen-free mouse room (12 hours light/12 hours dark; temperature, 22–24 °C), and received water ad libitum in the Animal Care Facility Service (Guangxi Medical University, China). The study protocol was approved by the ethics committee of the First Affiliated Hospital of Guangxi Medical University. All experimental procedures on mice were approved by the Committee on the Ethics of Animal Experiments of Guangxi Medical University. The study was carried out in strict accordance with the recommendations in the Guide for the Care and Use of Laboratory Animals of the National Institutes of Health.

### A mouse model of hepatic fibrosis

A total of 70 mice were randomly divided into hepatic fibrosis (n = 40, 8 mice/subgroup) and control groups (n = 30, 6 mice/subgroup). Each group was divided into 5 subgroups (0 week, 4^th^ week, 6^th^ week, 8^th^ week, and 12^th^ Week). To induce liver fibrosis, mice were intraperitoneally (IP) injected with 2mL/kg body weight of 20% CCl4 in olive oil (Shanghai Yangtze River Chemical Company, Shanghai, China) for 12 weeks. Mice were IP administered with olive oil (Sigma, USA) were used as control. Animals were sacrificed at 0 week, 4^th^ week, 6^th^ week, 8^th^ week, and 12^th^ week, 72 hours after the last injection. Livers and spleens were removed aseptically. Blood was harvested and plasma was prepared for analysis.

### Neutralization of IL-9 and IL-17

Mice were IP injected with 2 mL/kg body weight of 20% CCl4 (Shanghai Yangtze River Chemical Company, Shanghai, China). Day 0 was defined as the first day of CCl4 administration. Followed by injection, mice were injected IP with 100 μg IL-9 monoclonal antibody (R&D Systems, Inc. Minneapolis, MN. n = 10, anti-IL-9Ab group), or 100 μg isotype control immunoglobulin IgG (R&D, Systems, Inc. Minneapolis, MN. n = 10, IgG control group), or PBS (n = 10, PBS group) on day 0 and then with 100 μg two times a week until the 8^th^ week. Animals were sacrificed on 8^th^ week after CCl4 injection. The livers, spleens and plasma were collected.

### Histological and immunochemical assessment

The harvested liver tissues were fixed in 10% neutral buffered formalin and embedded in paraffin. Slices were prepared in 4 μm thickness and stained with hematoxylin/eosin (H&E) and Masson trichrome according to standard procedures. Without knowing the study design and data, two experienced pathologists assessed liver histology blindly by using light microscopy (Nikon Eclipse E800 Microscope, Kawasaki, Kanagawa, Japan). Ishak fibrosis score was used to define the degree of liver fibrosis. For immunohistochemistry, the sections were incubated with primary antibody (Sigma-Aldrich) in dilution of 1:300 (α-SMA), followed by incubation with streptavidin– peroxidase complex. Peroxidase conjugates were subsequently visualized using diaminobenzidine (DAB) solution. The sections were then counterstained with hematoxylin and mounted on a cover slip.

### Flow cytometry

#### Mouse

The splenic cells were gently dispersed through nylon mesh into a single-cell suspension, washed with RPMI 1640 (Gibco, USA). Single cell suspensions were prepared by lysing red blood cells with red blood cell lysing buffer (BD Biosciences, Vienna, Austria). The splenic mononuclear cells were then resuspended in RPMI 1640 medium with 10% FCS (Gibco, USA), stimulated with phorbol myristate acetate (PMA, 25 ng/ml, Sigma-Aldrich Poole, UK) and ionomycin (1 μg/ml, Sigma-Aldrich) in the presence of GolgiPlug (1 μl/10^6^cells, BD Biosciences) on a 24-well culture plate at 37 °C. After 5 h incubation, the cells were harvested and stained with PerCP-Cy5.5 conjugated anti-mouse CD4 antibody (BD Biosciences). Then cells were stained intracellularly with anti-IL-9-PE (eBioscience, San Diego, CA, USA), anti-IL-17-PE (eBioscience, USA), PE-conjugated anti-IL22 mouse antibody (eBioscience, USA) and anti-IFN-γ-Alexa-Fluor®488 (BD Biosciences) mouse antibody after fixation and permeabilization (BD Biosciences).

#### Human

Peripheral blood mononuclear cells (PBMC) were isolated from venous blood by ficoll separation using Ficoll-PaqueTM plus solution (Amersham Biosciences, Piscataway, NJ). Prior to flow cytometry, PBMC were washed in RPMI 1640 culture medium supplemented with 10% FCS, L-glutamine, penicillin/streptomycin and HEPES buffer (Cambrex, Invitrogen/Gibco, USA). The cells were incubated with FITC anti-human CD4 antibody (eBioscience, San Diego, CA) in room temperature for 20 min. After the surface staining, The cells were intracellularly stained with anti-human IL-9-PE (eBioscience, USA), anti-human IL-17-PE (eBioscience, USA), PE-conjugated anti-human IL22 (eBioscience, San Diego, CA, USA) and anti-human IFN-γ-Alexa-Fluor®488 (BD Biosciences) antibody after fixation and permeabilization. To deduct non-specific or background staining, we used isotype-matched antibodies as control.

Stained cells were further analyzed by FACS-Calibur flow cytometer (BD Bioscience) and FlowJo software 7.6.1 (Tristar, El Segundo, CA, USA). Th9, Th17, Th1 cells were defined as IL-9^+^ CD4^+^, IL-17^+^CD4^+^ and IFN-γ^+^CD4^+^ T cells respectively.

### Real-time PCR

Total RNA was extracted from homogenized liver tissues by using TRIZOL kit (Invitrogen, USA), and then reverse transcripted into cDNA with Reverse Transcription kit (Ferma, CA) according to the manufacturer’s instructions. Real time PCR was performed using SYBR Green (Invitrogen, USA) and ABI 7500 Sequence Detection System (Applied Biosystems, Foster City, CA). After an initial denaturation step for 3 min at 94 °C, a three-step cycling procedure (denaturation at 94 °C for 30 sec, annealing at 60 °C for 30 sec, and extension at 72 °C for 60 sec) was used for 35 cycles. The primer sets specific for transcripts of genes were as follows: mouse IL-9 (Forward) 5′-CCT TGC CTC TGT TTT GCT CTT C-3′ and (Reverse) 5′-GCT GCA TTT TGA CGG TGG ACT-3′; IL-17A, (Forward) 5′-GTG TCT CTG ATG CTG TTG-3′ and (Reverse) 5′-AAC GGT TGA GGT AGT CTG-3′; IFN-γ, (Forward) 5′-CTC AAG TGG CAT AGA TGT GGA AG-3′and (Reverse) 5′-GCT GGA CCT GTG GGT TGT TGA-3′; TNF-α, (Forward) 5′-AGT CCG GGC AGG TCT ACT TT-3′ and (Reverse) 5′-TTG GAC CCT GAG CCA TAA TC-3′; IL-6, (Forward) 5′-ACA GAA GGA GTG GCT AAG GAC C-3′ and (Reverse) 5′-TAG GCA TAA CGC ACT AGG TTT-3′; IL-4, (Forward) 5′-AGC AGT TCC ACA GGC ACA AG-3′ and (Reverse) 5′-AGC AGT TCC ACA GGC ACA AG-3′; IL-21, (Forward) 5′-CAC AGA CTA ACA TGC CCT TCA T-3′ and (Reverse) 5′-GAA TCT TCA CTT CCG TGT GTT C-3′;TGF-β1, (Forward) 5′-TGA GTG GCT TTT TGA CG-3′ and (Reverse) 5′-ACT TCC AAC CCA GGT CCT TC-3′; β-actin, (Forward) 5′-AAT TCC ATC ATG AAG TGT GA-3′ and (Reverse) 5′-ACT CCT GCT TGC TGA TCC AC-3′. The relative gene expression was normalized to the level of β-actin transcript and quantified by the ΔΔCT method using 7500 System Sequence Detection software (Applied Biosystems, CA, USA). All reactions were performed in duplicates for each sample.

### Cytokine assay

The plasma concentrations of IL-9 and IL-17 in mice were measured by Quantikine Mouse IL-9, IL-17A immunoassay (eBioscience, USA). The plasma concentrations of IFN-γ, TGF-β1, IL-6, TNF-α in mice were determined by ELISA kits (Boster, China), according to the manufacturer’s instructions. The sensitivity of ELISA kits for IL-9, IL-17A, IFN-γ, TGF-β1, IL-6, IL-4, IL-21 TNF-α was 5, 5, 7, 7, 7 and 4 pg/ml, respectively without detecting of any cross-reactivity. All samples were measured in triplicates. The HBV DNA load was determined using an ABI Prism 7000 Genetic Analyzer.

### Statistical analysis

Data were expressed as the mean ± standard deviation (SD). Two group comparisons were carried out using Student’s t test or Mann–Whitney U test when appropriated. One-way ANOVA test was used for multiple comparisons. Correlations were determined by Pearson’s correlation test. All data were analyzed using SPSS 16.0 software (SPSS Inc, Chicago, IL, USA). A two-sided p value of less than 0.05 indicated statistical significance.

## Additional Information

**How to cite this article**: Qin, S.-y. *et al.* A deleterious role for Th9/IL-9 in hepatic fibrogenesis. *Sci. Rep.* **6**, 18694; doi: 10.1038/srep18694 (2016).

## Supplementary Material

Supplementary Information

## Figures and Tables

**Table 1:** Clinical characteristics of the subjects enrolled in the study.

Group	HC	CHB	HBV-LC
Subjects	20	30	40
Male	12	18	30
Female	8	12	10
Age(years)	35.5(24–45)	34(18–71)	48.5(27–71)
ALT(U/L)	23.5(10–39)	286(55–889)	27.5(11–124)
AST(U/L)	24.5(12–36)	166(25–1138)	38.5(14–250)
TBIL(μmol/L)	8.45(3.8–16.3)	28.95(6.0–400.1)	22.65(6.7–468.8)
Child-Pugh(A/B/C)	ND	ND	14/15/7
HBeAg positive	0	20	18
HBV-DNA(copies/ml)			
1 × 10^3^−1 × 10^5^ copies/ml	0	8	31
>1 × 10^5^ copies/ml	0	12	9

Data are shown as median and range. HC, healthy control; CHB, chronic hepatitis B patients; HBV-LC, HBV associated liver cirrhosis patients. ND, not determined.
