# Supplementary material for: A deleterious role for Th9/IL-9 in hepatic fibrogenesis
Source: Sci Rep. 2016 Jan 5;6:18694. doi: 10.1038/srep18694 (PMC4700496; doi:10.1038/srep18694)
Supplement: Supplementary Information [file srep18694-s1.doc]

**Supplement materials**

**A deleterious role for Th9/IL-9 in hepatic fibrogenesis**

Shan-yu Qin^1^*, Dong-hong Lu^1^*, Xiao-yun Guo^1^*, Wei Luo^1^, Bang-li Hu^1^, Xiao-li Huang^1^, Mei Chen^1^, Jia-xu Wang^1^, Shi-Jjia Ma^1^, Xian-wen Yang^1^, Hai-xing Jiang1§, You Zhou^2,3^§

^1^ Department of Gastroenterology, the First Affiliated Hospital of Guangxi Medical University, Nanning 530021, China

^2^ Systems Immunity University Research Institute, Cardiff University School of Medicine, Heath Park, Cardiff, UK

^3^ Minerva Foundation Institute for Medical Research, Helsinki, Finland

Corresponding author:

Hai-xing Jiang, Ph.D.,

Clinical Medicine College of Guangxi Medical University

Nanning 530021 (China). E-mails: [gxjianghx@163.com](mailto:gxjianghx@163.com)

You Zhou, Ph.D.

Systems Immunity University Research Institute, Cardiff University School of Medicine, Heath Park, Cardiff CF14 4XN, Cardiff. Email: [you.zhou@helsinki.fi](mailto:you.zhou@helsinki.fi), yoyo.biooo@gmail.com


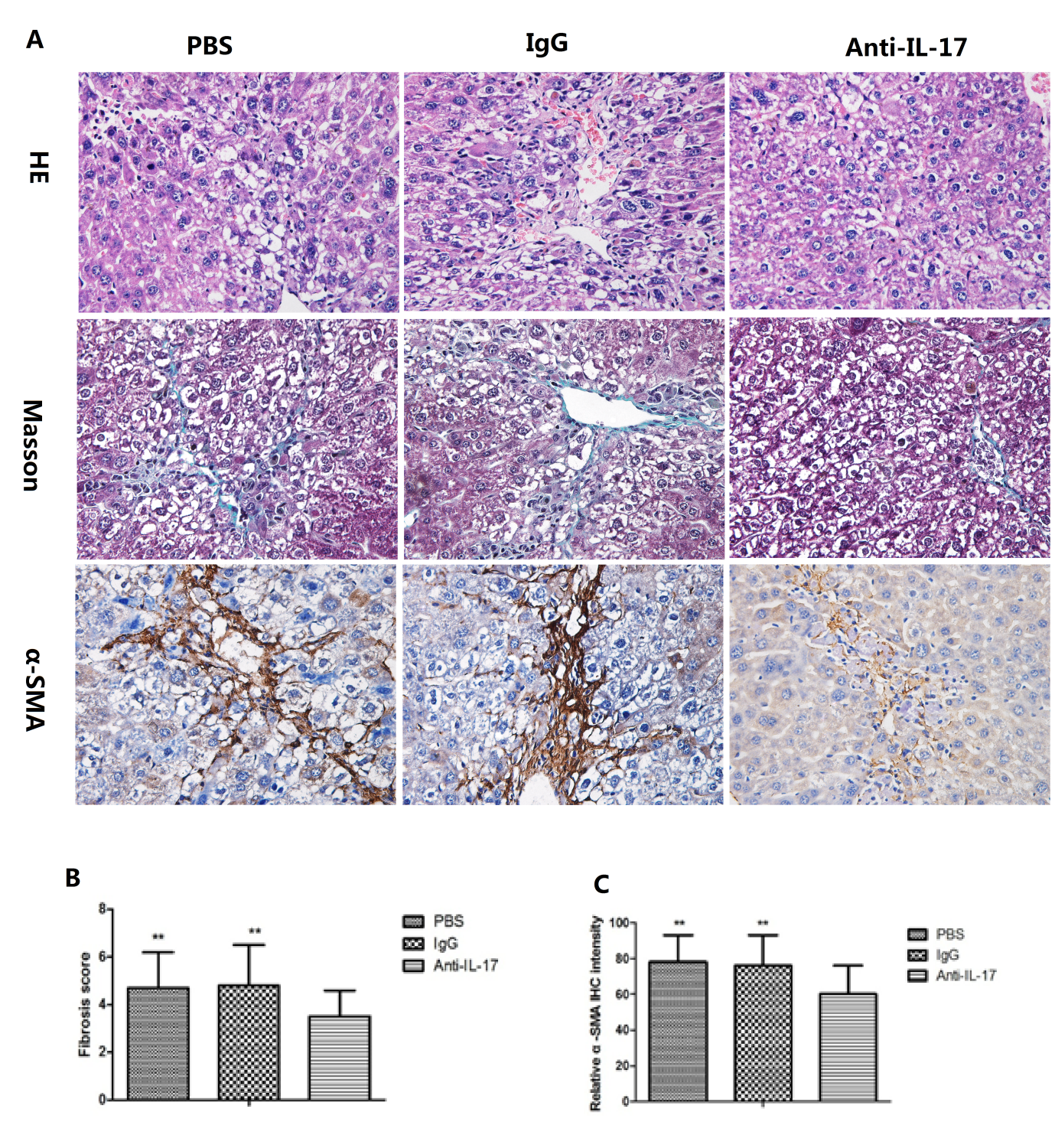


Figure S1: anti-IL-17Ab attenuated the severity of hepatic fibrosis. (A) Histology was assessed by H&E staining and fibrillar collagen deposition was evaluated by Masson staining (original magnification, ×400). (B) Comparison of Ishak fibrosis score between anti-IL-17Ab, PBS and IgG treated groups. (C) Morphometric quantitation of α-SMA expression between anti-IL-17Ab, PBS and IgG treated groups. **p<0.01 compared to those in IgG control and PBS groups. Data are mean ± SD (n=8).


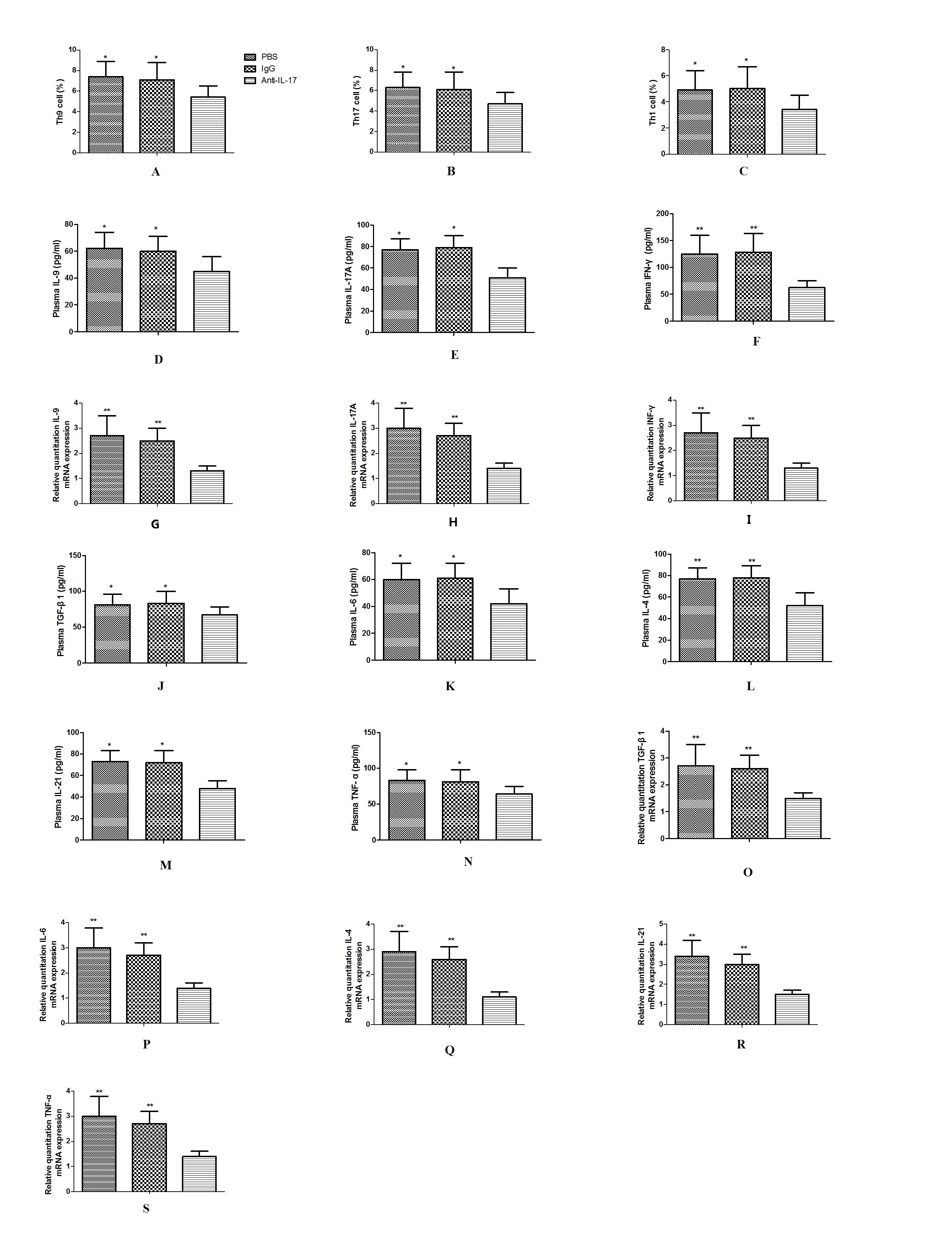


Figure S2: anti-IL-17Ab reduced splenic percentages of Th9, Th17 and Th1 cells and plasma secretion and liver expression of the relevant cytokines in mice with liver fibrosis. (A-C) The percentages of Th9, Th17 and Th1 cells in anti-IL-17Ab, IgG and PBS treated groups. (D-F) Plasma levels of IL-9, IL-17A and IFN-γ in anti-IL-17Ab, IgG and PBS treated groups. (G-I) The liver mRNA levels of IL-9, IL-17A and IFN-γ in anti-IL-17Ab, IgG control and PBS groups. (J-N) The plasma levels of TGF- β1, IL-6, IL-4, IL-21 and TNF-α in anti-IL-17Ab, IgG and PBS treated groups. (O-S) The liver mRNA levels of TGF-β, IL-6, IL-4, IL-21 and TNF-α in anti-IL-17Ab, IgG and PBS treated groups. ***p*< 0.01 compared to those in IgG control and PBS groups. Data are mean ± SD (n=8).
